# Retrospective Motion Correction in Multishot MRI using Generative Adversarial Network

**DOI:** 10.1038/s41598-020-61705-9

**Published:** 2020-03-16

**Authors:** Muhammad Usman, Siddique Latif, Muhammad Asim, Byoung-Dai Lee, Junaid Qadir

**Affiliations:** 1 Information Technology University (ITU)-Punjab, Lahore, 54700 Pakistan; 2 Center for Artificial Intelligence in Medicine and Imaging, HealthHub Co. Ltd., Seoul, 06524 South Korea; 3 University of Southern Queensland, Springfield, 4300 Australia; 4 Kyonggi University, Suwon, 16227 South Korea; 5 Department of Computer Science & Engineering, Seoul National University, Seoul, 08826 South Korea; 6 Distributed Sensing Systems Group, Data61, CSIRO, Pullenvale Queensland, 4069 Australia

**Keywords:** Computer science, Information technology

## Abstract

Multishot Magnetic Resonance Imaging (MRI) is a promising data acquisition technique that can produce a high-resolution image with relatively less data acquisition time than the standard spin echo. The downside of multishot MRI is that it is very sensitive to subject motion and even small levels of motion during the scan can produce artifacts in the final magnetic resonance (MR) image, which may result in a misdiagnosis. Numerous efforts have focused on addressing this issue; however, all of these proposals are limited in terms of how much motion they can correct and require excessive computational time. In this paper, we propose a novel generative adversarial network (GAN)-based conjugate gradient SENSE (CG-SENSE) reconstruction framework for motion correction in multishot MRI. First CG-SENSE reconstruction is employed to reconstruct an image from the motion-corrupted *k*-space data and then the GAN-based proposed framework is applied to correct the motion artifacts. The proposed method has been rigorously evaluated on synthetically corrupted data on varying degrees of motion, numbers of shots, and encoding trajectories. Our analyses (both quantitative as well as qualitative/visual analysis) establish that the proposed method is robust and reduces several-fold the computational time reported by the current state-of-the-art technique.

## Introduction

Magnetic resonance imaging (MRI) is a safe, non-ionizing, and non-invasive imaging modality that provides high resolution and excellent contrast of soft tissues. It has emerged as a powerful and effective technique for early diagnosis of many common but potentially treatable diseases including stroke, cancer, and ischemic heart disease. Despite these advantages, the prolonged data acquisition time of MRI causes many difficulties in its clinical applications, and various research efforts have been proposed in response to expedite the data acquisition process including the use of parallel imaging (PI)^1^, compressed sensing (CS)^2^, and echo-planar imaging (EPI)^3^.

In single-shot echo-planar imaging (EPI), all the *k*-space data necessary to reconstruct the final magnetic resonance (MR) image is acquired in a single excitation pulse. It significantly accelerates the data acquisition time and minimizes the possibility of motion artifacts in MR images^4,5^. However, MR images reconstructed using single-shot EPI suffers from low resolution and susceptibility artifacts. To overcome these limitations, segmented EPI or multishot MRI is used^6^, which is a compromise between echo-planar and standard spin-echo imaging. It significantly reduces the demands on gradient performance and allows the in-plane spatial resolution to be improved to a level comparable to that of standard spin echo pulse sequences^7^. In multi-shot MRI, the *k*-space data is acquired in using a large number of shots at different time instances to obtain the high-resolution volumetric image. As a result, the image may be severely degraded due to subject motion between consecutive shots. This makes the multishot sequences very sensitive to shot-to-shot variabilities caused by the motion.

On the basis of the source of motion, motion in MRI is classified into two categories. *Rigid motion* is caused by the movement of a solid part of body, in which deformation is zero or so small it can be neglected, such as arm, knee, and head motion, while *non-rigid* motion arises from those parts of body, which does not retain any consistent shape, like cardiac motion^8^. The rigid motion produces acute artifacts^9^, which may cause suboptimal image quality, especially intra-brain scan where the contribution of rigid motion is more significant in contrast to non-rigid motion. Subsequently, it may negatively impact radiologic interpretation^10^, which affects patient safety and enhances the medico-legal risks related to the interpretation of motion degraded images. Therefore, motion correction techniques are considered as an imperative part of MRI reconstruction processes^11^.

Previously, the problem of motion correction has been solved mostly in an iterative manner^12^, which is time-consuming as well as computationally extensive. Researchers are now increasingly interested in leveraging recent advances in deep learning (DL) for improving the state-of-the-art performances in the healthcare^13,14^. In particular, the use of generative adversarial networks (GANs)^15^ is interesting due to its capability of generating data without the explicit modelling of the probability density function and also due to its robustness to over-fitting. The adversarial loss brought by the discriminator formulated in GANs provides a clever way of forcing the generative network to produce sharp and highly continuous data that can be useful for motion correction in MRI.

In this paper, we propose using a GAN-enhanced framework to correct rigid motion in multishot MRI. We focus on brain structural scans due to their frequent use and significance in clinical settings^16^. This work is the extension of our previous preliminary work^17^, where we empirically showed the suitability of GAN for motion correction in multishot MRI. In particular, we are proposing a GAN-based conjugate gradient (CG) SENSE^18^ reconstruction model to correct the motion in multishot MRI. The proposed techniques involve the use of CG-SENSE for the reconstruction of the motion-corrupted multishot *k*-space data, which is then fed to a GAN to produce an artifact-free image. The proposed technique is effective in reducing motion artifacts and in reducing the computation time, which makes our technique attractive for clinical applications. We have validated our method on publicly available data by changing various parameters of multishot MRI—such as the amount of motion, the number of shots, and the encoding trajectories—with our results showing impressive performance in producing artifact-free image across different parameters in significantly less reconstruction time as compared to traditional iterative techniques.

## Background and Related Work

MRI is highly sensitive to subject motion during the *k*-space data acquisition, which can reduce image quality significantly by inducing motion artifacts. Such artifacts, particularly those produced by rigid motion, are widely observed in multishot MR images during the clinical examination^16^. The application of motion correction techniques during or after the reconstruction process, therefore, becomes essential to ensure that an artifact-free image is obtained.

Retrospective motion correction (RMC) techniques are applied to the rigid motion correction^19,20^. RMC techniques are post-processing techniques employed after the acquisition of the *k*-space data, while the data is acquired without considering the potential motion^8^ and the object motion is estimated from acquired *k*-space data. Researchers have proposed a number of RMC-based method for rigid motion correction. For instance, Bydder *et al*.^21^ studied the inconsistencies of *k*-space caused by subject motion using parallel imaging (PI) technique. The inconsistent data is discarded and replaced with consistent data generated by the PI technique to compensate the motion artifacts. This method produces an image with fewer motion artifacts, albeit at a cost of a lower signal to noise ratio (SNR).

Loktyushin *et al*.^22^ proposed a joint reconstruction and motion correction technique to iteratively search for motion trajectory. Gradient-based optimization approach has been opted to efficiently explore the search space. The same authors extended their work in a subsequent work^23^ by disintegrating the image into small windows that contain local rigid motion and used their own forward model to construct an objective function that optimizes the unknown motion parameters. Similarly, Cordero *et al*.^24^ proposed the use of a forward model to correct motion artifacts. However, this technique utilises the full reconstruction inverse to integrate the information of multi-coils for estimation and correction of motion. In another study^25^, authors extended their framework to correct three-dimensional motion (i.e., in-plane and through-plane motion). Through the plane, the motion is corrected by sampling the slices in an overlapped manner.

Conventional techniques such as those just discussed estimate the motion iteratively, which makes them computationally extensive and time-consuming and therefore unsuitable for use in time-critical medical applications. Recent advancements in the field of DL has facilitated significant advance in the medical imaging research community—but very limited attempts have been made for motion correction in MRI. Loktyushin *et al*.^26^ studied the performance of convolution neural network (CNN) for retrospective motion correction in MR images and proposed training of a model for learning a mapping from motion-corrupted data to motion-free images. The study indicated the potential application of deep neural networks (DNNs) to solve the motion problem in MRI; however, the study did not provide detailed quantitative results or a detailed investigation of the utilized technique. Similarly, Duffy *et al*.^27^ used CNN to correct motion-corrupted MR images. The work has been compared with traditional Gaussian smoothing^28^ and significant improvement has been reported but comparison with the advanced state-of-the-art iterative motion correction techniques was unaccounted. Importantly, previous DL-based *motion correction* studies have not exploited GANs despite the fact that GANs have shown excellent performance in MRI reconstruction in particular^29,30^, and more broadly in modelling natural images^31,32^ and in biomedical image analysis^33^.

In our previous work^17^, we proposed the use of GAN for multishot MRI motion correction. This work presented the preliminary results on motion correction by notably reducing the computational time. However, the study did not perform a detailed performance evaluation of the proposed multishot MRI framework against the various parameters such as *the number of shots* and *the encoding trajectories*. Building on our previous work, we propose an adversarial CG-SENSE reconstruction framework for the correction of the motion. A detailed analysis of the proposed framework has been presented with respect to different parameters of multishot imaging such as *the levels of motion, the number of shots*, and *the encoding trajectories*.

## Methodology

**Figure 1:**
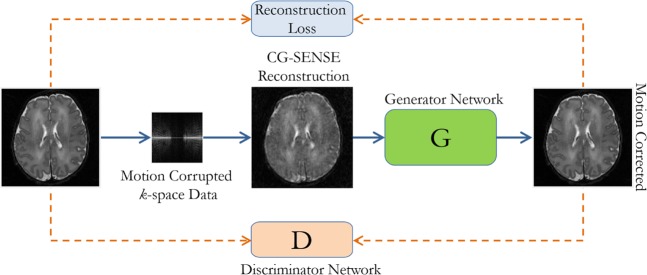
The proposed motion correction framework for multishot MRI, where CG-SENSE is used to reconstruct motion-corrupted images, and the generator network of the GAN, in conjunction with the discriminator network, is tasked with motion correction (Figure Credit: Latif *et al*.^17^).

In our proposed method, reconstruction and motion correction are performed, independently. Standard CG-SENSE is employed to reconstruct *k*-space data which provides a motion-corrupted image in the spatial domain. Motion-corrupted images are given to the GAN for reduction of motion artifacts in the second stage. Figure 1 shows the overall proposed architecture.

### Motion model for multishot MRI

In Multishot MRI, *k*-space data is acquired in multiple shots (i.e., 2, 4 or 8 shots) in order to cover the whole *k*-space. The MRI scanners capture Fourier coefficients along encoding trajectories that are directed by the gradient shapes of the MRI sequence. For generating motion-corrupted data, we opted the same model as followed by^24,26^, originally proposed by Batchelor *et al*.^19^

**Figure 2:**
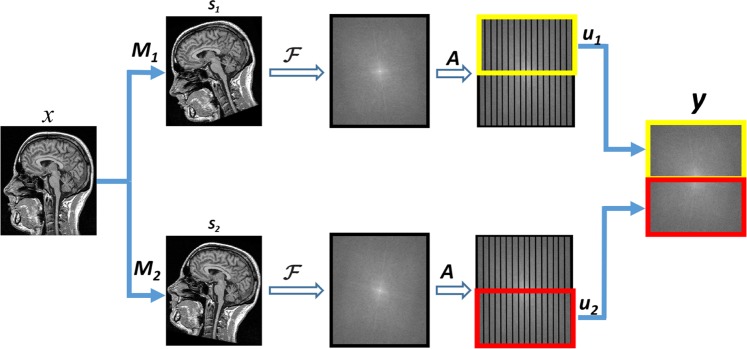
Forward motion corruption model (in 2D) for single coil and two shots MRI: *x* is the motion-free image; *M*_*s*_ is responsible for introducing the motion in particular shot; *F* and *A* employs DFT and sampling; and *u*_*s*_ extracts the *k*-space segment for each shot.

In this model, motion *M*_*s*_ is introduced for each *s*^*th*^ shot in a motion-free image *x*. Subsequently, Fourier transform *F* and sampling matrix *A* is applied to achieve the *k*-space representation. Finally, the segment *u*_*s*_ of *k*-space is extracted for each shot and eventually, all the segments are combined to obtain the full *k*-space data. Mathematically, it can be written as:

1 $$y=\mathop{\sum }\limits_{s=1}^{N}x{M}_{s}FA{u}_{s}$$

where, *N* represents the number of shots, *M*_*s*_ the translation as well rotational motion for *s*^*th*^ shot, and *y* the motion-corrupted *k*-space data. Figure 2 shows the forward motion model for single coil and two shots.

### Conjugate gradient SENSE (CG-SENSE) reconstruction

In our proposed technique, we employ CG-SENSE reconstruction technique to reconstruct motion-corrupted *k*-space data. It utilises conjugate gradient (CG)^34^ algorithm to efficiently solve the SENSE equations^35^, which relates the gradient encoding, sensitivities and aliased images to unaliased ones. CG-SENSE algorithm relates the object to be imaged *x*_*m*_, the encoding matrix *E* and the acquired *k*-space data *y* as follows:

2 $$E{x}_{m}=y$$

The acquired data *y* has size *n*_*c*_*n*_*k*_, where *n*_*c*_ and *n*_*k*_ are the number of coils and the number of sampled positions in *k*-space, respectively. The size of reconstructed image *x*_*m*_ is *N*^2^, while *N* is the matrix size of the image. The spatial encoding information of gradients and coil sensitivities, is presented by the encoding matrix *E*.

To solve Eq. (2), *E* has to be inverted, which is a difficult task due to its large size. CG algorithm is used to iteratively solve Eq. (2) for the unaliased image, due to its fast convergence compared to other methods^36^. To facilitate the formulation of the CG-SENSE reconstruction, another matrix *Z* is introduced to inverse the encoding as follows:

3 $$ZE={I}_{d}$$

where, *Z* and *I*_*d*_ represents the reconstruction matrix and the identity matrix, respectively. Multiplying both sides of Eq. (2) by the *F* matrix results into an unaliased image which can be described as:

4 $${x}_{m}=Zy$$

The reconstruction matrix *Z* can be computed by employing Moore-Penrose inversion:

5 $$Z={({E}^{H}E)}^{-1}{E}^{H}$$

Now the set of equations can be solved without finding the inverse of the *E* matrix by employing CG algorithm. To efficiently perform the CG-SENSE reconstruction process pre-conditioning is performed for better initial estimation of *x*^36^.

### Generative adversarial framework

GANs^15^ are latent variable generative models that learn via an adversarial process to produce realistic samples from some latent variable code. It includes a generator *G* and a discriminator *D* which play the following two-player min-max game:

6 $$\mathop{\min }\limits_{G}\ \mathop{\max }\limits_{D}\quad {{\rm{P}}}_{x}[{\rm{\log }}\,(D(x))]+{{\rm{P}}}_{z}[{\rm{\log }}\,(1-D(G(z)))]$$

In a simple vanilla GAN, the generator *G* maps the latent vectors drawn from some known prior *p*_*z*_ (simple distribution e.g. Gaussian) to the sample space. The discriminator *D* is tasked with differentiating between samples generated *G*(*z*) (fake) and data samples (real).

Here, we use conditional GAN^37^, where instead of random samples, *G* is fed corrupted MRI images *x*_*m*_ and is trained to produce motion corrected image *x*_*c*_. The adversarial training loss ${\boldsymbol{\mathscr{L}}}_{{\rm{adv}}}$ for *G* is defined as

7 $${\boldsymbol{\mathscr{L}}}_{{\rm{adv}}}={\rm{\log }}\,(1-D(G({x}_{m})))$$

To facilitate the generator, in addition to the adversarial loss, we also incorporate data mismatch term.

8 $${\boldsymbol{\mathscr{L}}}_{{\rm{data}}}=\parallel {x}_{c}-G({x}_{m}){\parallel }_{2}$$

Adversarial training encourages the network to produce sharp images, which is of crucial importance in MRI imaging, whereas data mismatch loss forces the network to correctly map degraded images to the original ones. Thus the final loss for *G*, dubbed generator, is a weighted sum of ${\boldsymbol{\mathscr{L}}}_{{\rm{data}}}$ and ${\boldsymbol{\mathscr{L}}}_{{\rm{adv}}}$ .

9 $$\boldsymbol{\mathscr{L}}={\boldsymbol{\mathscr{L}}}_{{\rm{data}}}+\lambda {\boldsymbol{\mathscr{L}}}_{{\rm{adv}}}$$

where *λ* is a hyper-parameter that controls the weight of each loss term. As training progresses, *G* and *D* are trained iteratively.

## Experimental Setup

### Dataset

For the evaluation of the proposed method, publicly available data is utilized. The data is obtained from the MICCAI Challenge on Multimodal Brain Tumor Segmentation (BraTS) organized by B. Menze, A. Jakab, S. Bauer, M. Reyes, M. Prastawa, and K. Van Leemput^38–42^. The challenge database contains fully anonymized images from the following institutions: ETH Zurich, University of Bern, University of Debrecen, and University of Utah. We followed the data usage agreement provided by BraTS (https://www.med.upenn.edu/sbia/brats2018/registration.html) and all the experiments were carried out in accordance with relevant guidelines and regulations.

We used T2 FLAIR images of high grade (HG) tumor scans; the BraTS 2015 dataset contains 274 HG scans of different subjects. We divided the scans into three subsets including training, validation, and testing sets that contain 191, 25 and 58 scans respectively. Each scan in the dataset has already been normalized into a standard size (i.e., 240 × 240 × 255). However, we further refined the scan while extracting each slice by cropping it from the center and resizing it into 128 × 128 size. The blank slices in each scan are discarded and a total of 37627, 4875, and 11484 images for training, validation, and testing are produced, respectively. Images of BraTS dataset are considered as motion-free images and motion is introduced by employing the model described in Section 2. The same perturbation technique has been employed in other works^25,26^. As BraTS contains spatial domain images, we used a reference scan to estimate the coil sensitivity maps as in Allison *et al*.^43^. For our work, we produce data with varying degrees of angular motion, number of shots, and trajectories to validate the robustness of our proposed technique.

### Model architecture

**Figure 3:**
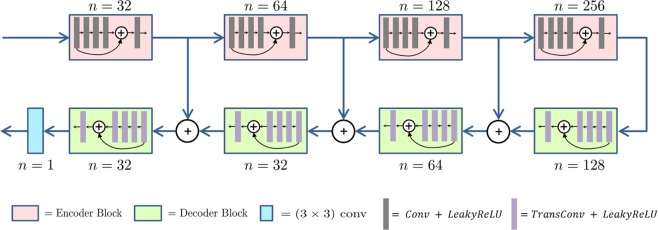
U-Net like Model Architecture used as Generator and Discriminator in GAN.

We adopt a U-Net like architecture (shown in Fig. 3) because of its recent success in image restoration task^2,44^. It has an hour-glass like structure that involves encoder and decoder networks. The encoder bottlenecks the important information from the corrupted image by reducing the motion artifacts and the decoder is responsible to restore motion free image. In our paper, the encoder consists of convolutions blocks, where each block consists of convolutional layers following by non-linear activation; decoder blocks are composed of transposed convolution layers.

The U-Net architecture also contains symmetric skip connections from encoder blocks to the decoder blocks. It helps to recover fine details for better image restoration: encoder learns to compress image into the high-level features necessary for image restoration, but may remove fine details along with the corruptions, whereas the skip connections from encoder to decoder transfer low-level features from the encoding path to the decoding path to recover the details of the image. In addition to these skip connections, we employ residual connections^45^ inside each encoder and decoder block like Milletari *et al*.^46^. These residual connections along with skip connections allow efficient gradient flow, which helps in alleviating issues such as vanishing gradients and slow convergence.

The high-level model architecture is described in Fig. 3. Each encoder block consists of 5 convolution layers, each with *n* feature maps except for the layer in the middle with *n*/2 feature maps. Padding is employed to keep the dimension of feature maps same inside each block. We set the strides equal to 1 for all layers except the first one, where we choose it to be 2. This stride 2 convolution serves to down-sample feature maps using a learned kernel. Inside each encoder block, a residual connection is used between the first layer and the last layer. Decoder block has the same structure as the encoder except that we replace all the convolutional layers with transposed convolutions and use a stride of 2 at the last layer instead of the first layer. Here stride 2 transposed convolution serves to up-sample the feature maps along the U-Net architecture. The discriminator is exactly the same as the encoder part of the generator.

### Model training

We train our network using training data and validation data was used for parameters selection. We evaluated the model in the testing phase using held out data i.e., testing set. We selected the best value of *γ* by evaluating the model on different values (0.1, 0.2, 0.4, 0.5, 0.6, 0.8, 1.0) of *γ*. The value of *γ* giving the best results on the validation set is used for evaluations in testing phase. For all experiments in this paper, we achieve best results using *γ* > 0.5. We optimized the model using RMSProp with the learning rate being 1 × 10^−4^ until convergence. We use a batch size of 16. For each update of *G*, we update *D* twice. We pre-train the generator *G* using Adam optimizer with same learning rate and batch size. This allows the training of *G* to converge faster.

### Quantifying parameters

We used the following three parameters to measure the quantitative performance of our proposed framework.

#### Peak signal to noise ratio (PSNR)

It is the ratio of maximum possible value (power) of a signal and the power of distorting noise that affects the quality of its representation. We calculated the PSNR of our resultant images by using formulation as follows:

10 $$PSNR=20{{\rm{\log }}}_{10}\left(\frac{\max (r)}{\sqrt{MSE}}\right)$$

where *MSE* is mean square error which can be calculated as

11 $$MSE=\left(\frac{1}{mn}\right)\mathop{\sum }\limits_{i=0}^{1-m}\mathop{\sum }\limits_{j=0}^{1-n}{| | r(i,j)-x(i,j)| | }^{2}$$

where *r* represents the reference image, *x* denotes the reconstructed image, *m* and *n* are the numbers of rows and columns of the reconstructed image, and max function computes the maximum value.

#### Structural similarity index (SSIM)

This is a very common method for predicting the quality of a reconstructed image by checking its similarity with the reference image. The SSIM index is calculated on various windows of an image^47^ which can be formulated as

12 $$SSIM(r,x)=\frac{(2{\mu }_{r}{\mu }_{x}+{c}_{1})(2{\sigma }_{rx}+{c}_{2})}{({\mu }_{r}^{2}+{\mu }_{x}^{2}+{c}_{1})({\sigma }_{r}^{2}+{\sigma }_{x}^{2}+{c}_{2})}$$

Where,

- *r* is the reference image
- *x* is the reconstructed image
- *μ*_*r*_ is the mean value of reference image
- *μ*_*x*_ is the mean value of reconstructed image
- ${\sigma }_{r}^{2}$ is the variance of *r*
- ${\sigma }_{x}^{2}$ is the variance of *x*
- *σ*_*rx*_ is the covariance of *r* and *x*;

#### Artifact power (AP)

It represents the level of artifacts in any given image with reference to the ground truth. AP can be defined as^48^

13 $$AP=\frac{\sum {| | r(i,j)| -| x| | }^{2}}{\sum {| r(i,j)| }^{2}}$$

Higher the value of the AP, the more the artifacts; therefore, the reduction of APs is attempted to achieve an artifact-free image.

## Results and Discussion

In this section, we have performed a detailed investigation of our proposed technique for the reconstruction of motion-free images in the presence of varying levels of motion, number of shots, and encoding trajectories. Since motion is corrected in the spatial domain which allows the solution to be employed to any kind of motion and encoding/sampling scheme. However, considering the immense range of potential sampling trajectories, acquisition orderings, patterns of motion and number of shots, we restrict our evaluation to a limited set of encoding trajectories, number shots, and degrees of rotational motion. Further, we also perform a comparison of our results with the state-of-the-art technique by Cordero *et al*.^25^, which has been selected for comparison because of the closeness of its reconstruction process to the one proposed here. Particularly, Cordero *et al*. also used the same forward model of the acquisition process to add perturbation into the motion free image. Most importantly, they demonstrated a significant improvement in terms of reconstruction error as compared to the previous state-of-the-art technique^26^. For validation, we used peak signal to noise ratio (PSNR), structural similarity index (SSIM), and artifact power (AP) as quantification parameters.

### Effect of the levels of motion

Our work is focused on rigid motion correction, specifically for intra-brain scan head motion^9^, which is mostly rotational motion and it causes austere effects in the reconstructed image. Therefore, to evaluate the effect of motion, different rotational motion artifacts have been introduced into motion-free images with 16-shots and random trajectory. The motion-corrupted *k*-space data has been reconstructed using CG-SENSE (without motion correction) and then fed to the adversarial network, which is tasked to generate motion-free images. Table 1 summarizes the average results obtained for varying degrees of rotational motion (Δ*θ* = {2°, 5°, 8°, 10°, 12°, 14°}) on test data. It can be noted from Table 1 that the proposed framework shows excellent performance for a small amount of motion by capturing the underlying statistical properties of MR images, and recover sharp and excellent images. However, with the increase in the amount of motion, a smooth decay in the performance of model is observed, as expected, because with higher degree of inter-scan motion (i.e., 14°) MRI scans get severely degraded and it becomes very difficult to recover the motion free image.

**Table 1:** Performance metrics of our approach on different amount of motion with 16-shots and random trajectory.

Degree of motion	2°	5°	8°	10°	12°	14°
Peak signal to noise ratio (PSNR)	32.31 ± 1.93	31.57 ± 1.57	30.89 ± 1.85	28.18 ± 1.92	27.85 ± 1.63	27.25 ± 1.74
Structural similarity index (SSIM)	0.96 ± 0.061	0.96 ± 0.067	0.94 ± 0.047	0.92 ± 0.052	0.91 ± 0.068	0.90 ± 0.063
Artifact power (AP)	2.47 × 10^−3^ ± 0.13 × 10^−3^	4.52 × 10^−3^ ± 0.17 × 10^−3^	6.57 × 10^−3^± 0.29 × 10^−3^	7.31 × 10^−3^ ± 0.27 × 10^−3^	8.08 × 10^−3^ ± 0.29 × 10^−3^	9.10 × 10^−3^ ± 0.38 × 10^−3^

**Figure 4:**
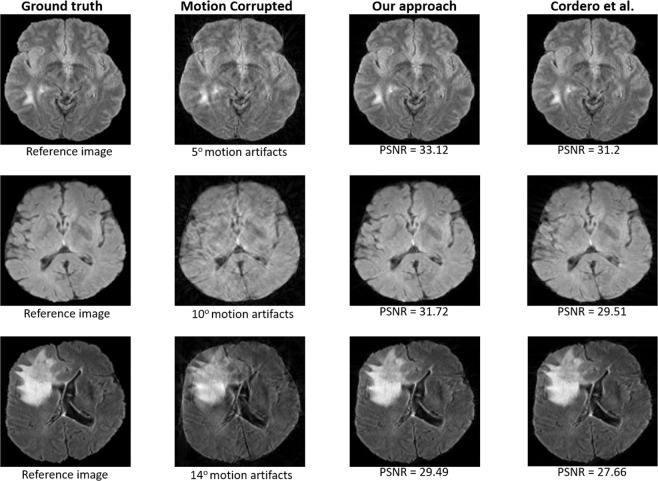
Resultant images produced by our approach compared to those produced by Cordero *et al*.^25^ for Δ*θ* = {5°, 10°, 14°} with 16-shot and random trajectory.

Moreover, the performance of our technique is better than the previous state-of-the-art iterative technique^24^ for higher levels of motion (i.e., Δ*θ* = 14°) (see Fig. 4). For a small amount of motion, the approach of Cordero *et al*.^24^ performs slightly better in terms of AP, however, the long computational time restrains its efficiency.

### Influence of the number of shots

**Figure 5:**
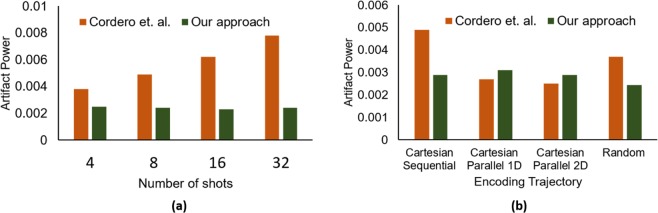
Comparison of our framework with the state of the art iterative technique^24^ for fifty randomly selected test images in terms of the (**a**) number of shots and (**b**) encoding trajectories.

In this experiment, we investigate the performance of the proposed framework for different number of shots. We generated motion-corrupted data for various number of shots, (i.e., S = {2,4,8,16,32,64,128}) with *five degree* of motion and *the random trajectory*. We trained our model individually for each *number of shots* and evaluated the performance. The results are summarized in Table 2, which describes the mean values of results obtained on all the test scans. It can be seen from Table 2 that the network is able to learn the artifact pattern and provides significantly promising results for all the number of shots. Encouragingly, our network produces sharp images with high values of PSNR and SSIM even for a higher number of shots. In contrast, state-of-the-art iterative technique^24^ were only able to correct the motion for lower number of shots effectively. In Fig. 5(a) a snippet of performance comparison against the different number of shots has been shown on fifty randomly selected test images with 2° of motion. It can be witnessed that our method has similar performance for each number of shots, while the conventional technique^24^ gradually reduces performance with the increase in the number of shots. For higher number of shots (*S* > = 32), the convergence of such iterative techniques^24,26^ becomes very difficult. In our case, motion is corrected in the spatial domain after the full reconstruction of the motion-corrupted image, which enables the adversarial network to correct the motion artifacts in the image domain without encountering such convergence challenges.

**Table 2:** Performance metrics of our approach for varying shots at 5 degree.

Number of Shots	2	4	8	16	32	64	128
Peak signal to noise ratio (PSNR)	31.82 ± 2.23	31.92 ± 2.55	31.55 ± 1.89	31.57 ± 2.21	31.93 ± 1.92	32.02 ± 2.08	32.08 ± 1.76
Structural similarity index (SSIM)	0.95 ± 0.057	0.96 ± 0.053	0.96 ± 0.048	0.96 ± 0.045	0.96 ± 0.043	0.96 ± 0.041	0.96 ± 0.042
Artifact power (AP)	4.52 × 10^−3^ ± 0.29°× 10^−3^	4.42 × 10^−3^ ± 0.26 × 10^−3^	4.32 × 10^−3^ ± 0.25 × 10^−3^	4.13 × 10^−3^ ± 0.18 × 10^−3^	3.56 × 10^−3^ ± 0.18 × 10^−3^	3.59 × 10^−3^ ± 0.23 × 10^−3^	3.46 × 10^−3^ ± 0.19 × 10^−3^

**Figure 6:**
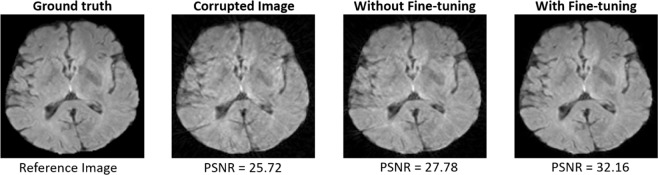
Results from model, without and with fine-tuning, have been shown on a motion-corrupted image (with 16 number of shots).

We also evaluated the robustness of the proposed model. We trained the model using a higher number of shots and testing is performed using a lower number of shots. The model slightly improves the PSNR of the reconstructed image, which is not suitable for real-time applications as shown in Fig. 6. This is due to the fact that the motion artifacts produced in the images reconstructed from the lower number of shots are different than the artifacts produced from the higher number of shots. However, initializing the model with the weights of higher number of shots and fine-tuning with the lower number of shots helps improve convergence. Therefore, we followed the same method for each number of shots to expedite the convergence.

### Influence of the encoding trajectory

**Figure 7:**
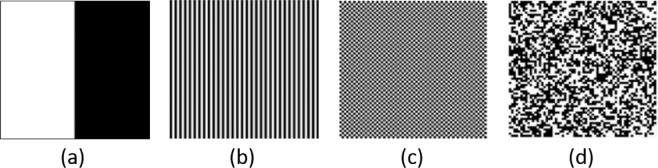
Encoding strategies used for experiments, depicted for S = 2 shots: (**a**) Cartesian sequential, (**b**) Cartesian parallel 1D, (**c**) Cartesian parallel 2D, (**d**) Random; samples corresponding to one of the shots are in white, otherwise samples are in black.

From the vast range of trajectories, we restricted ourselves to the four trajectories (as shown in Fig. 7) to validate the performance of the proposed framework. The motion-corrupted data of each encoding trajectory is generated with eight number of shots (*S* = 8) and a relative rotation of Δ*θ* = 5° is assumed between shots. We first performed the full reconstruction of motion-corrupted *k*-space data for each encoding trajectory and then trained GANs with the resultant motion artifact-corrupted images, individually for each trajectory.

Table 3 describes the mean results of our proposed framework for each encoding trajectory. The results show that our approach performs significantly well for all the encoding trajectories. However, it can be noted through close observation that the performance of the proposed technique is slightly better for the *random trajectory* since the random trajectory is least affected by the motion. The same reasoning can be applied for slightly degraded performance for Cartesian sequential trajectory as this trajectory is most affected by the motion artifacts. On the other hand, the iterative technique^24^ vigorously changes its performances against different encoding trajectories as depicted in Fig. 5(b) where we compare the proposed technique with the solution proposed by Cordero *et al*.^24^ for different encoding trajectories on fifty randomly selected test images with 2 degrees of motion. For *Cartesian sequential trajectory*, this technique takes an extraordinarily large number of iterations to reach the convergence, while the proposed technique has universal acceptance and it can be employed to any encoding trajectory.

**Table 3:** Performance of our approach for different trajectories of multishot MR imaging for eight number of shot (*S* = 8) and 5° of motion.

Sampling trajectory	Cartesian sequential	Cartesian parallel 1D	Cartesian parallel 2D	Random
Peak signal to noise ratio (PSNR)	30.11 ± 2.10	30.14 ± 1.80	30.72 ± 1.53	31.55 ± 1.89
Structural similarity index (SSIM)	0.95 ± 0.057	0.95 ± 0.047	0.95 ± 0.57	0.96 ± 0.062
Artifact power (AP)	5.51 × 10^−3^ ± 0.44 × 10^−3^	5.425 × 10^−3^ ± 0.37 × 10^−3^	5.025 × 10^−3^ ± 0.25 × 10^−3^	4.32 × 10^−3^ ± 0.30 × 10^−3^

### Computational time analysis

In this section, we present the results of the comparison of the computational time of our technique with the state-of-the-art iterative technique^24^. To keep our analysis fair, we performed the motion correction of same motion-corrupted *k*-space data on the same hardware—specifically, an Intel^®^ Core^TM^ i3-2120 CPU with 3.5GHz speed with 16GB of memory and NVIDIA^®^ Quadro M5000 Graphic Processing Unit (GPU) with 8GB GDDR5 memory—by employing both techniques. Since the proposed technique involves two steps (CG-SENSE reconstruction and motion correction), we added the reconstruction and motion correction time to compute the total computational time. Table 4 provides a relative summary of the computational time analysis of our technique compared with the solution proposed by Cordero *et al*.^24^ for a varying number of shots for 50 randomly selected test images. It can be seen that our technique is several times faster than the previous iterative approach^24^. The previous technique is an iterative method that first iteratively estimates the motion and then corrects for that motion, which needs extra computational time. With the increase in the number of shots, it becomes difficult to estimate the motion between two consecutive shots, subsequently, it further increases the time required to correct the motion for higher numbers of shots. Moreover, changing the encoding trajectory also significantly affects the computational performance of the conventional iterative technique^24^. Alternatively, in our proposed technique, motion correction is independent of the reconstruction process and it is performed after full reconstruction of *k*-space data. Therefore, the motion correction for all the number of shots takes the same computational time. However, the CG-SENSE reconstruction takes more time for the higher number of shots, which slightly increases the overall motion corrected reconstruction time (see Table 4). In Table 5, we summarize the computational time of our technique and iterative technique^24^, against different levels of motion. The time required to correct for motion in our technique is not dependent upon the amount of motion, therefore, it remains the same for all levels of motion. Alternatively, the conventional technique takes longer time to estimate the higher amount of motion, thus it takes more time to correct such motion.

**Table 4:** Comparing computational time of our approach with the current state-of-art technique^24^ against different number of shots.

Number of shots	4	8	16	32	64	128
Cordero *et al*.	9.00s	12.80s	14.59s	27.83s	50.07s	89.19s
Our approach	0.23s	0.28s	0.34s	0.48s	0.79s	1.40s

**Table 5:** Comparing computational time of our approach with the current state-of-art technique^24^ for various levels of motion.

Degree of motion	5°	8°	10°	12°	14°
Cordero *et al*.	25.23s	39.27s	57.93s	134.27s	152.03s
Our approach	0.28s	0.28s	0.28s	0.28s	0.28s

## Conclusion

We introduced a flexible yet robust retrospective motion correction technique that employs generative adversarial networks (GANs) to correct motion artifacts in multishot Magnetic Resonance Imaging (MRI). This work is an extension of our previous preliminary work, where we empirically showed the suitability of GAN for motion correction in multishot MRI. The proposed technique first performs the full reconstruction of motion-corrupted *k*-space data and then the resultant artifact-affected image is fed into the deep generative networks that learns the mapping from motion artifact-affected images to the artifacts free images. Our GAN based framework significantly reduces the motion artifacts without any prior estimation of motion during the data acquisition or reconstruction process in contrast to the previous iterative methods. Such a parameter-free technique can be employed to any encoding scheme without introducing modifications in the acquisition sequence. To validate our method, we carried out comprehensive experimentation by varying different parameters, such as different levels of motion, the number of shots, and encoding schemes, of multishot MRI. Based on the results, we demonstrated that the performance of the proposed technique is more robust against these parameters and it also reduced the computational time significantly in contrast to the state-of-the-art techniques. Future plans include the extension of framework to perform end-to-end learning using the generative network from motion-corrupted under-sampled coil information (*k*-space data) to artifacts free image.
